# Genome annotation of *Caenorhabditis briggsae* by TEC-RED identifies new exons, paralogs, and conserved and novel operons

**DOI:** 10.1093/g3journal/jkac101

**Published:** 2022-04-29

**Authors:** Nikita Jhaveri, Wouter van den Berg, Byung Joon Hwang, Hans-Michael Muller, Paul W Sternberg, Bhagwati P Gupta

**Affiliations:** Department of Biology, McMaster University, Hamilton, ON L8S 4K1, Canada; Department of Biology, McMaster University, Hamilton, ON L8S 4K1, Canada; Division of Biology and Biological Engineering, California Institute of Technology, Pasadena, CA 91125, USA; Division of Biology and Biological Engineering, California Institute of Technology, Pasadena, CA 91125, USA; Division of Biology and Biological Engineering, California Institute of Technology, Pasadena, CA 91125, USA; Department of Biology, McMaster University, Hamilton, ON L8S 4K1, Canada

**Keywords:** nematode, *C. briggsae*, *trans*-splicing, spliced leader, operons, paralog, genome annotation

## Abstract

The nematode *Caenorhabditis briggsae* is routinely used in comparative and evolutionary studies involving its well-known cousin *Caenorhabditis elegans*. The *C. briggsae* genome sequence has accelerated research by facilitating the generation of new resources, tools, and functional studies of genes. While substantial progress has been made in predicting genes and start sites, experimental evidence is still lacking in many cases. Here, we report an improved annotation of the *C. briggsae* genome using the *trans*-spliced exon coupled RNA end determination technique. In addition to identifying the 5′ ends of expressed genes, we have discovered operons and paralogs. In summary, our analysis yielded 10,243 unique 5′ end sequence tags with matches in the *C. briggsae* genome. Of these, 6,395 were found to represent 4,252 unique genes along with 362 paralogs and 52 previously unknown exons. These genes included 14 that are exclusively trans-spliced in *C. briggsae* when compared with *C. elegans* orthologs. A major contribution of this study is the identification of 492 high confidence operons, of which two-thirds are fully supported by tags. In addition, 2 SL1-type operons were discovered. Interestingly, comparisons with *C. elegans* showed that only 40% of operons are conserved. Of the remaining operons, 73 are novel, including 12 that entirely lack orthologs in *C. elegans*. Further analysis revealed that 4 of the 12 novel operons are conserved in *Caenorhabditis nigoni.* Altogether, the work described here has significantly advanced our understanding of the *C. briggsae* system and serves as a rich resource to aid biological studies involving this species.

## Introduction

Nematodes are a mainstay in fundamental biological research. While most work has been based on *Caenorhabditiselegans* over the last half a century since its proposed role as a model organism (Brenner 1974), the close relative *Caenorhabditisbriggsae* offers many of the same advantages in carrying out studies. Despite diverging roughly 20–30 million years ago (Cutter 2008), the 2 species exhibit similar behavioral, developmental, and morphological processes including a hermaphroditic mode of reproduction (Gupta *et al.* 2007). Moreover, many of the experimental techniques and protocols developed to manipulate *C. elegans* can be adopted to *C. briggsae* with minimal to no modification (Baird and Chamberlin 2006; Gupta *et al.* 2007). These features make *C. briggsae–C. elegans* an ideal pair for comparative and evolutionary studies.

The genome of *C. briggsae* was sequenced many years ago and revealed extensive genomic and genic conservation with *C. elegans* (Stein *et al.* 2003). Subsequent work reported the assembly of genomic fragments into chromosomes and improved gene predictions (Hillier *et al.* 2007; Ross *et al.* 2011). While a diverse array of techniques have been applied to improve the annotation of the *C. elegans* genome (Hwang *et al.* 2004; Spieth and Lawson 2006; Hillier *et al.* 2009; Salehi-Ashtiani *et al.* 2009; Allen *et al.* 2011), a similar approach is lacking for *C. briggsae*. The current *C. briggsae* genome annotation is largely based on homology with the *C. elegans* genome. More analysis that uses experimental data gathered directly from *C. briggsae* is needed to improve gene identification and gene models. To this end, we used *trans*-spliced exon coupled RNA end determination (TEC-RED) (Hwang *et al.* 2004), a technique based on exploiting the phenomenon of spliced leader (SL) *trans*-splicing which has been observed in nematodes and several other phyla including platyhelminths, chordates, and trypanosomes (Lasda and Blumenthal 2011).

The advantage of TEC-RED compared with other genome annotation techniques like EST (Marra *et al.* 1998) and SAGE (Velculescu *et al.* 1995) is that it is capable of identifying transcripts of most expressed genes, and uniquely allows for the identification of 5′ transcript start sites and alternative transcripts with different 5′ ends of a gene. The approach is based on 2 principles: (1) a short sequence from the 5′ end of a transcript can be used to uniquely identify the initiation site of the transcript and (2) the 5′ ends of most mRNAs are spliced to common leader sequences known as SL sequences. The SL *trans*-splicing process involves replacing the outron of a pre-mRNA with a 22 nucleotide SL sequence donated by a 100-nucleotide small ribonucleoprotein (Blumenthal 2005; Allen *et al.* 2011). *C.elegans* and *C. briggsae* both have 2 types of SL sequences: SL1 and SL2 (Qian and Zhang 2008; Blumenthal *et al.* 2014).

We recovered well over 120,000 5′ end tags from sequencing reactions representing 10,243 unique tags (7,234 for SL1; 3,009 for SL2) with matches in the *C. briggsae* genome. The tags were analyzed using WormBase release WS276 to map unique hits in the genome, resulting in the identification of a total of 4,252 genes. Other tags with high confidence hits to unannotated regions or to multiple locations of the genome identified 52 novel exons and 362 paralog genes, respectively. The novel exons could either represent previously unknown genes or new exons of existing genes. The paralogs define 133 sets of 2 or more genes. Of these sets, 21 were confirmed as exact matches with known paralogs in WormBase. The rest could potentially be new paralogous pairs that need further validation. While the majority of the genes discovered by tags confirmed 5′ ends of genes listed in WormBase, there are many for which 5′ ends indicated by tags differ from current gene models, suggesting the need to revise existing annotations.

A comparison of the splicing pattern of *C. briggsae* genes with *C. elegans* revealed some changes. Specifically, 14 genes are spliced to leader sequences in *C. briggsae* but their *C. elegans* orthologs lack such splicing information. We also investigated the presence of operons. It was reported earlier that 96% of *C. elegans* operons are conserved in *C. briggsae* based on collinearity (Stein *et al.* 2003). Our analysis revealed a total of 1,198 operons including 492 for which splicing identities of 2 or more genes are reported in this study. Of these operons, 333 are fully supported by tags. Comparison of the latter with *C. elegans* revealed that 40% are conserved, the largest of which is composed of 7 genes. Another 38% are termed partially conserved since gene sets do not fully correspond to any of the operons in *C. elegans*. The remaining are novel, i.e. consisting of divergent genes as well as genes whose *C. elegans* orthologs are not reported in operons. Of the divergent operons, 4 were found to be conserved in a closely related sister species, *C.nigoni*. Lastly, 2 SL1-type operons have been identified. Overall, the results presented in this study have substantially improved the annotation of the *C. briggsae* genome by identifying the 5′ ends of a large number of genes as well as discovering novel operons, new exons, and paralogs. The findings strengthen the utility of *C. briggsae* as a model organism and serve as a platform to accelerate comparative and evolutionary studies involving nematodes and other metazoans.

## Materials and methods

### Generation of tags

We followed the protocol described earlier for *C. elegans* (Hwang *et al.* 2004). Briefly, the steps involved purification of poly(A) RNA from the wild-type *AF16* mixed stage animals, RT-PCR to generate cDNA, amplification of cDNAs using biotin-attached primers homologous to SL1 and SL2 sequences carrying mismatches to create *Bpm*I restriction enzyme site (Supplementary Tables 1–3), digestion of amplified cDNAs using *Bpm*I to produce short fragments (termed “5′ tags”), ligation of tags to adaptor DNA sequences, and sequential ligation of DNA to create concatenated products. The ligated DNA pieces were cloned into a vector and sequenced.

### 5′-Tag sequence analysis and exon identification

We wrote several Perl scripts to analyze the tags and genes. A flowchart is provided in Supplementary Fig. 1. Briefly, tags were collected and assigned unique tag IDs. Tag locations in the genome were determined by comparing the tag sequence to WS176 and WS276 genome files, where orientation and chromosome location for each tag was noted. Subsequently the splice sequence for each tag was obtained by finding the 7 bases directly upstream of each location where a tag matched on the genome.

The criteria to identify tag matches to exonic regions were described earlier (Hwang *et al.* 2004). These included “same orientation of the tag as that of the corresponding exon,” “distance to the first ATG,” “a minimum distance to the nearest in-frame stop codon,” and “presence of a splice acceptor sequence following the tag.” The latter was scored on how well they fit the consensus splice site “TTTTCAG” (Blumenthal and Steward 1997). In cases where tags had multiple matches, we applied stricter splice acceptor site criteria. Perfect consensus sequence was given the highest weight. Sites having mismatches were assigned lower weights with priority given to conserved bases. While this approach resulted in most tags identifying unique exons, a small number still showed multiple matches and were used to search for potential paralogs (see below).

**Fig. 1.**
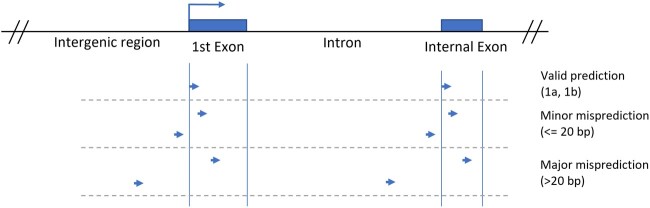
Representative model of locations of tag sequences within the genome. Three broad categories of matches are valid prediction (termed 1a and 1b), minor misprediction, and major misprediction.

Each tag was used to find the nearest ATG of an open reading frame, i.e. the proposed start of a coding sequence (CDS). This ATG location was compared with known coordinates of start sites of nearest exons as annotated in WS176 and WS276 genome annotation (gff3) files. This was done using coordinates of annotated CDS. Two broad categories of exon matches were identified based on tags that had unique matches: (1) where the 5′ end corresponded to the start of a known exon (first exon: 1a, internal exon: 1b) and (2) matches for which the 5′ end differed from a nearest exon (Fig. 1). Depending on the distance between the 5′ end and the exon, the second category of matches was further divided into 2 subcategories. These consisted of exons that were either within 20 bp from the 5′ end (“minor misprediction”) or further away (“major misprediction”). The major misprediction subcategory also includes matches where 5′ ends were more than 3 kb away and may define brand new exons of existing genes as well as potentially new, previously unknown genes.

### Manual curation of genes

We found that 75 tag-matched genomic regions in the WS276 gff3 file had no known genes/exons within 3 kb downstream of the matched ATG. The surrounding chromosomal regions of these matches were confirmed by manually searching the WormBase genome browser for presence of annotated exons. Of the 75, 21 were found to be false positives due to incorrect script calls. Two were excluded from analysis because the genes are not assigned to any chromosomes. The remaining 52 matches may represent novel exons.

### Analysis of intergenic regions and operons

The distance between 2 genes, termed “intergenic region” (IGR) was determined based on the distance from the end of the 3′ UTR of an upstream gene to 5′ start of the CDS of the immediate downstream gene. Graphs were generated using Graphpad Prism 7.0 and Microsoft Excel. Genes having IGR >5,000 bp (257) were excluded from the analysis. For pairs of genes where the second gene is located within the first gene, IGR length is calculated as a negative value. Intercistronic regions (ICRs) were calculated in the same manner. The ICR analysis was done only for genes identified to be part of an operon.

We assigned genes to an operon in *C. briggsae* using criteria established in *C. elegans* that is based on the SL identity and IGR between subsequent genes (Blumenthal 2005). In brief, all genes *trans*-spliced with SL2 or SL1/SL2 and present downstream of an SL1-spliced gene were categorized into a single operon model along with the upstream SL1-spliced gene. We based our assumption of genes being in an operon together on this pattern of SL sequences. If the splicing of the first upstream gene was unknown, the operon models were termed “nontag-supported” whereas those models in which the identity of the first upstream gene was known were termed “tag-supported.” We compared the “tag-supported” operon models with those in *C. elegans* (WormBase) to determine how well operons are conserved. Based on the conservation of genes, the “tag-supported” operons were classified into Exact match, Partial match, and Novel.

We confirmed the splicing of *Cbr-rpb-6* by reverse transcription followed by polymerase chain reaction (RT-PCR) amplification using SL1 and SL2 primers and a sequence-specific down primer GL1764 (GTTGAAGTTGTTCGGTGG). The examination of PCR-amplified products on an agarose gel showed a strong signal for the SL1 primer and a faint signal for the SL2 primer (both same size). The SL1 primer-amplified piece was confirmed by sequencing.

We examined the enrichment of germline genes in *C. briggsae* high confidence operons. For this, *C. elegans* orthologs were identified and researched for association with germline function (Wang *et al.* 2009). The significance of overlap was tested by the hypergeometric probability test. Next, to identify processes related to genes in operons, gene ontology (GO) (Ashburner *et al.* 2000) analysis was carried out for all operon genes. A similar analysis was conducted for genes present in *C. elegans* operons using a published dataset (Allen *et al.* 2011).

### Paralog analysis

A total of 203 tags had multiple hits in the genome. Since many of these consisted of overlapping sequences, we retained only the longest tags. This filtering step narrowed down the count to 158. The genes identified by these tags were compared with annotated paralogs in WormBase. The matches allowed us to place the predicted paralogs into 3 different categories. Genes that were paired as paralogs with newly discovered exons were excluded.

### Uniquely spliced *C. briggsae* genes

To identify the genes that are *trans*-spliced in *C. briggsae* but not in *C. elegans*, we used datasets published by 2 different groups that together constitute the most complete collection of *trans*-spliced genes in *C. elegans* (Allen *et al.* 2011; Tourasse *et al.* 2017). Initial comparisons with the Allen *et al.* dataset revealed 198 genes that are present only in our analysis. The number was further reduced to 14 genes when compared with the Tourasse *et al.* study (Supplementary File 3).

## Results

### Overview of the TEC-RED method in *C. briggsae*

To implement the TEC-RED approach to identify transcripts, we first isolated *C. briggsae* mRNAs containing an SL1 or SL2 sequence at their 5′ ends. A total of 121,189 5′ tags (91,733 for mRNA with an SL1 and 29,456 for mRNA with an SL2 SL sequence) were recovered from DNA sequencing reactions. These tags represent 14,678 different sequences, of which 10,400 (71%) are for SL1 and 4,278 (29%) for SL2 sites. More than two-thirds of all tags found matches in the genome (10,243, 70%), of which 46% are unique, i.e. matching only once and others matching multiple times (Table 1). The proportions were similar for both SL categories, demonstrating no bias in the experimental protocol. The remaining 4,435 tags (30%) had no match, for which there might be several reasons. One, our search criteria was strict. Since TEC-RED tags are very short (∼14 nucleotides), we did not allow for mismatches at the risk of getting too many nonspecific hits in the genome. Other possibilities include sequencing errors, gaps in the genome sequence, and incorrect sequence assembly.

**Table 1.** Overview of SL1 and SL2 5′ tags identified in the study.

	Total unique tags	Matches in genome	Unique hits	Multiple hits
All	14,678	10,243	4,753	5,490
SL1	10,400	7,234	3,281	3,953
SL2	4,278	3,009	1,472	1,537

### Exon validations and predictions in *C. briggsae* based on 5′ tag matches

**Fig. 2.**
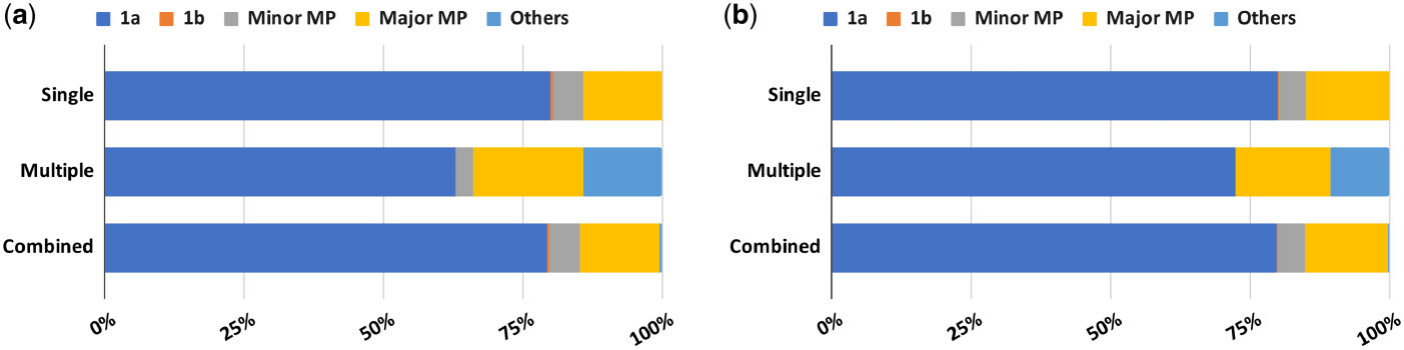
Proportion of tags belonging to different categories. The majority of SL1 (a) and SL2 tags (b) have single (unique) hits in the genome and belong to category 1a, i.e. predicted 5′ ends match with WormBase gene models. 1b: predicted 5′ end match with an internal exon. Minor MP, minor misprediction; Major MP, major misprediction; Others: mixed category of matches.

A total of 62.5% of all tags (6,395 of 10,243) matched to exonic regions and were retained for further analysis (Table 2). The remaining tag matches were excluded because they did not pass the search criteria (such as incorrect orientation, nonconsensus splicing site, etc.; see *Materials andMethods* for details). Next, we determined the locations of these tags relative to annotated exons in WormBase. Most of the tags (6,192, 96.8%) matched uniquely to 1 exon, with a small number having multiple matches (203, 3.2%) (Supplementary File 1). For both SL1 and SL2 tags, roughly 80% of the matches correspond to known 5′ ends of annotated genes (category 1a), providing support to existing gene models in WormBase. Less than 1% of the tags matched to internal exons (category 1b), suggesting an alternate 5′ end of the corresponding genes. The remaining tags identified start sites that differed from current WormBase gene models and were categorized as mispredicted genes. In most of these cases (roughly three-quarters of all mispredictions) the nearest exon was more than 20 bp away. This leads us to suggest that, particularly in these cases, existing gene models may need to be revised. These exons may define new 5′ ends of known genes as well as novel, previously unidentified genes. More experiments are needed to investigate these possibilities. As expected, both types of tags, i.e. with unique and multiple hits have a similar distribution of categories (Fig. 2; Supplementary File 1).

**Table 2.** Breakdown of tag matches into different categories.

Category of tag matches	SL1	SL2	Total
1a	3,537	1,542	5,079 (79.4%)
1b	20	3	23 (0.3%)
Minor misprediction	245	91	336 (5.2%)
Major misprediction	639	291	930 (14.5%)
Others	22	5	27 (0.4%)
Total	4,463	1,932	6,395

The numbers include both unique and multiple hits. Tag matches termed as “Others” are those that cannot be placed uniquely into any of the main categories.

### Identification of genes based on tag matches

Next, we compiled a list of *C. briggsae* genes based on exons identified by unique tags. A total of 4,252 genes were recovered by SL1 and SL2 tags (Supplementary File 2). Almost two-thirds of the genes (65%) are spliced with SL1 and 18% with SL2. Another 18% of exons matched with both SL1 and SL2 tags (SL1/SL2), suggesting the genes are part of hybrid operons (Allen *et al.* 2011) (Table 3; Supplementary File 2). Based on their genomic locations, these genes are roughly evenly distributed on the chromosomes except for Chr. X which had the lowest gene count. However, the trend was different for gene density with Chr. III being the densest chromosome and Chr. X the sparsest (Supplementary Table 4). Whether the uneven distribution is by chance or a characteristic of *trans*-spliced genes in *C. briggsae* remains to be seen. A tiny fraction of genes (0.1%) is located on unmapped genomic fragments.

**Table 3.** Breakdown of genes by spliced leader sequences.

Spliced leader type	Number of genes
SL1	2,750 (65%)
SL2	743 (18%)
SL1/SL2	759 (18%)
Total	4,252

The recovery of *C. briggsae* genes prompted us to examine evolutionary changes in *trans*-splicing. A comparison with *C. elegans* studies (Allen *et al.* 2011; Tourasse *et al.* 2017) revealed 14 genes that appear to be uniquely spliced to leader sequences in *C. briggsae* but not in *C. elegans* (Supplementary File 3).

Next, we searched for transcripts resulting from *cis*-splicing of the *C. briggsae* genes. Almost 95% of the curated genes (4,025 of 4,252) were found to be associated with unique tag sequences, i.e. 5′ ends matched to just 1 exon, providing support for the presence of a single transcript for these genes (Table 4). In the majority of cases (82%, 3,288 of 4,025), the tag-identified 5′ ends matched with a known first exon (category 1a tags). Less than 1% of the tags identify 5′ ends that match with internal exons (category 1b). The remaining genes (18%) consist of exons belonging to minor and major misprediction categories. The rest of the genes (5%, 227 of 4,252) identified by tags consist of those that produce multiple transcripts (Table 5). In 84% of these cases, at least one 5′ end identified by tags matched with the first exon (category 1a). Five of the genes were alternatively spliced using internal exons as the 5′ start site (category 1b). Most of the genes consisted of at least 1 major mispredicted exon, suggesting that genes with multiple splice variants require further validation.

**Table 4.** Genes supported by the presence of a single 5’ end (single transcript)

Category of matches	SL1	SL2	SL1/SL2	Total
1a	2,142 (65.2%)	558 (17.0%)	587 (17.9%)	3,287
1b	14 (87.5%)	1 (6.2%)	1 (6.2%)	16
Minor misprediction	146 (71.6%)	34 (16.7%)	24 (11.7%)	204
Major misprediction	357 (69%)	127 (24.6%)	33 (6.4%)	517
**Total**	**2,659**	**720**	**645**	**4,024**

Numbers refer to genes identified by SL1, SL2 and SL1/SL2 tags. Novel exons and potential paralogs are excluded.

**Table 5.** Genes supported by the presence of multiple 5’ ends.

Category of matches	SL1	SL2	SL1/SL2	Total
1a and 1b	2 (25%)	0	3 (75%)	5
1b	0	0	0	0
1a and minor misprediction	14 (36%)	7 (18%)	18 (46%)	39
1a and major misprediction	57 (39%)	14 (10%)	74 (51%)	145
Others	2 (33%)	0	4 (67%)	6
All mispredicted exons	16 (50%)	2 (6%)	14 (44%)	32
**Total**	**91**	**23**	**113**	**227**

Numbers refer to genes identified by SL1, SL2 and SL1/SL2 tags. Genes for which exons belong to multiple categories are grouped as ‘Others’. Novel exons and potential paralogs are excluded.

As mentioned previously, 203 tags had multiple matches in the genome. Further analysis narrowed down the set to 158 unique sequences (see *Materials and Methods*). We reasoned that these tags may represent paralogs and performed searches in WormBase. The analysis identified 133 potential paralog sets consisting of 362 genes. These sets fall into 3 distinct categories (Supplementary File 10). Paralogs that fully matched with WormBase annotation were termed “Exact Match” (21 paralogous sets, 42 genes). The other sets matched only partially or did not match to paralog sets recorded on WormBase (Partial Match: 66 sets, 174 genes; No Match: 46 sets, 146 genes). It is worth mentioning that about half of the genes in the No Match category have no paralogous information available, whereas the remaining half have paralogs in WormBase but these did not match with our analysis. To further validate the paralogous relationships, we determined chromosomal locations of the genes. Gene duplications arising from mechanisms such as slipped-strand mispairing can cause the creation of paralogous genes in adjacent stretches of sequence on the same chromosome (Levinson and Gutman 1987). In *C. elegans*, paralogs originating from gene duplications are more than twice as likely to be present on the same chromosome and tend to be located closely together (Semple and Wolfe 1999). Additionally, studies in humans and other higher eukaryotes have revealed that intergenic distances between paralogous genes are smaller than random gene pairs on the same chromosome (Ibn-Salem *et al.* 2017). Of the paralog sets identified in this study, 63% (84 sets) were present on the same chromosome including 35% (16 sets) that belong to the No Match category. The IGR analysis revealed that the distances in 5 cases are <10 kb (Supplementary Table 5), which is more than 500-times shorter than the average distance between a random pair of genes on the same chromosome (5.58 ± 0.89 Mb in *C. elegans*) (Lee and Sonnhammer 2003).

### Validations of TEC-RED-identified transcripts

**Fig. 3.**
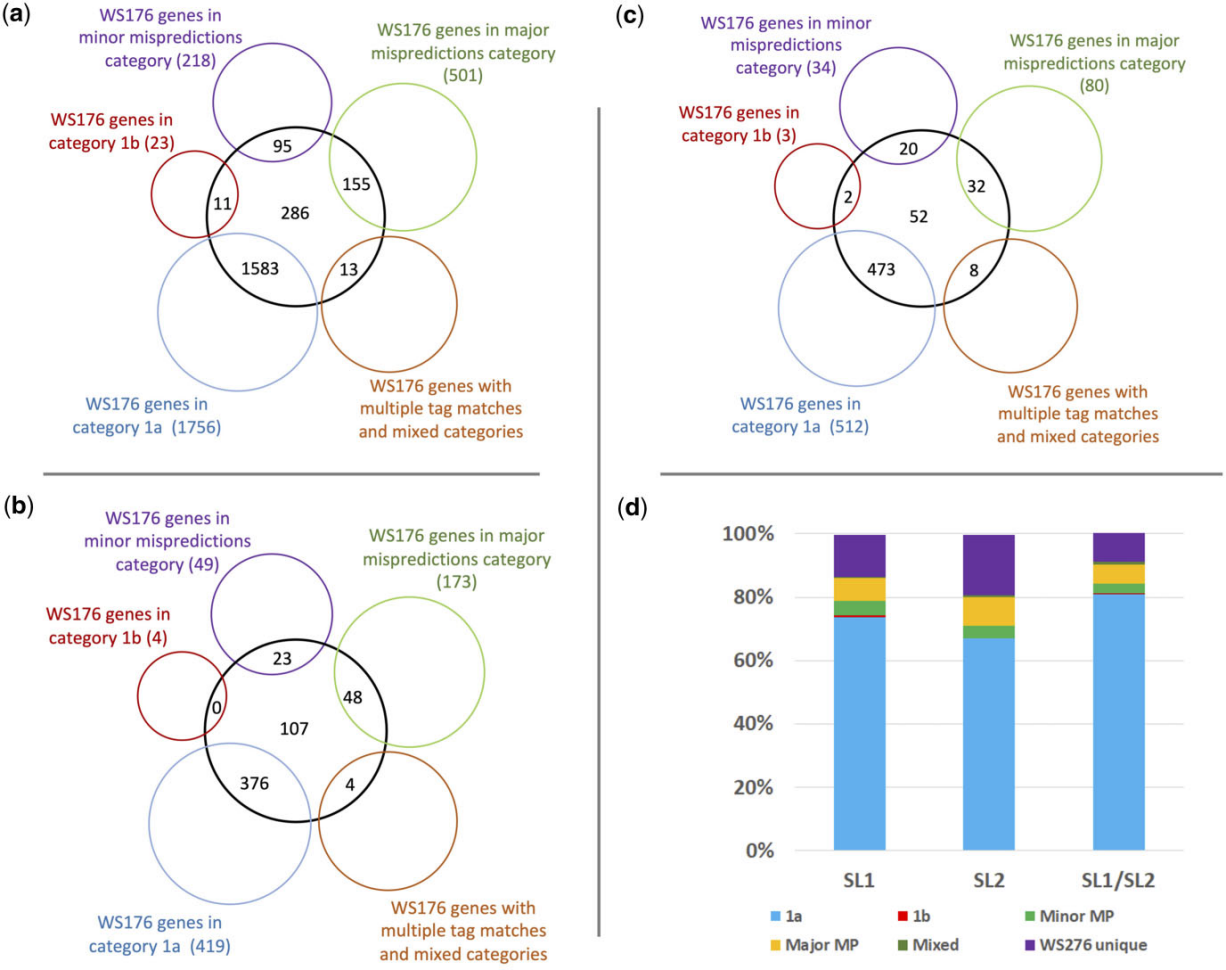
Reclassification of genes from WS176 categories to category 1a in WS276. Only single transcript genes were compared. Venn diagrams, with WS276 genes of category 1a in black circles and WS176 genes of various categories in colored circles. Numbers in overlapping circles represent genes of a given category in WS176 that are annotated as 1a type in WS276. Numbers in the middle of black circles (nonoverlapping) represent genes that are unique to WS276 analysis. a) 286 or 13.2% of SL1-spliced; b) 107 or 19.0% of SL2-spliced; c) 53 or 9% of SL1/SL2 hybrid-spliced] whereas those in brackets next to colored circles are total genes identified by tag searches in WS176. d) Histogram showing the proportion of genes with matching 5′ ends in WS276 (category 1a) that overlap with various categories in the WS176 analysis.

We took 3 different approaches to validate subsets of TEC-RED predictions with the goal of demonstrating the usefulness of the technique in improving gene identification and gene models. One approach involved comparing different categories of tag-identified exons between 2 WormBase releases. As described above, a significant number of exons are categorized as minor and major mispredictions (22%, 943 of 4,252; see Tables 4 and 5). We hypothesized that mispredicted exons may be confirmed with improvements in genome annotation. To test this hypothesis, category 1a of transcripts were compared with those reported in an older WormBase release (WS176). The analysis involved SL1 spliced transcripts belonging to category 1a (2,142) (Table 4). As expected, a vast majority of the genes (73.9%, 1,583) are in category 1a in both releases, providing support for these gene models (Fig. 3, a and d; Supplementary File 4). The next 2 largest categories consist of genes that are mispredicted (11.7%, 250 genes) and newly predicted, i.e. absent in WS176 (13.3%, 286 genes). Few genes (0.5%, 11) have start sites that correctly match with internal 5′ ends of internal exons. The rest (0.6%, 13 genes) could not be uniquely placed into a single category since these had multiple tag matches in the older annotation. Roughly, similar results were obtained by analyzing 1a category of SL2 spliced and SL1/SL2-spliced genes (182 genes, 115 genes, respectively) (Table 4 and Fig. 3, b–d; Supplementary File 4). Altogether, 858 annotation improvements are supported by our analysis. The demonstrated improvements in gene identification and genome annotation as observed in WS276 prove the accuracy of our 5′ start site determination method.

The second type of validation focused on a subset of the major misprediction category of genes whose 5′ ends mapped more than 3 kb away from nearest exons. Most of these (94%, 49 of 52) are in IGRs (Supplementary File 5). Thirty-seven percent (19 of 52) of the exons are supported by RNA sequencing reads (WormBase), providing proof of accuracy to our method (Supplementary Fig. 2). These novel exons are likely to either belong to nearby existing genes or define brand new genes.

The last set of validations consisted of comparisons with *C. elegans* gene models. In this case, category 1b of single and multiple transcripts (Tables 4 and 5, respectively) were manually examined. The results showed that 38% of newly discovered 5′ ends (6 single transcript and 2 multiple transcripts) are supported by *C. elegans* orthologs (Supplementary Fig. 3 and File 6), providing further support to our analysis. We took a similar approach to analyze a subset of transcripts in the major mispredictions category. Of the 10% of such predictions that were tested, 34% (17 of 50) are supported by WormBase *C. elegans* gene models. With this success rate, another 115 of the remaining single transcript genes of the major misprediction category are likely to be validated. Overall, the 5′ tag analysis serves as a rich resource to improve the *C. briggsae* genome annotation.

### Discovery of operons

The identification of genes based on unique tag matches in *C. briggsae* allowed us to search for operons. In *C. elegans* it has been shown that the first gene in an operon is SL1 spliced (Conrad *et al.* 1991), whereas downstream genes are spliced either with SL2, SL2 variants or both SL1 and SL2 (Blumenthal 2005). An operon that contains at least 1 gene spliced with both SL1 and SL2 is considered a “hybrid operon.” Ultimately, global analysis of *trans*-splicing in *C. briggsae* will reveal all operons and operon genes.

Our data suggest the existence of up to 1,198 *C. briggsae* operons (Table 6; Supplementary File 7). These include 333 operons that are fully supported by tags, i.e. we were able to determine the splicing pattern of every gene, with operons ranging from 2 to 7 genes The remaining 865 operons (ranging between 2 and 6 genes) are categorized as “Predicted operons” since the splicing identity of the first gene in these cases remains to be determined. In this set, the predicted operons that contain 3 or more genes (159) are large enough to be labeled as bona fide operons. Added together with the 333 fully supported operons, this allows us to report at least 492 operons in *C. briggsae* with sufficient certainty.

**Table 6.** Breakdown of *C. briggsae* operons based on the number of genes present.

	No. of operons	Operons consisting of					
		2 genes	3 genes	4 genes	5 genes	6 genes	7 genes
Tag-supported operons	333	261	54	15	2	0	1^*a*^
Predicted operons	865	706	125	26	7	1	0

Operons are placed into 2 broad categories, those consisting entirely of genes with known spliced leader sequences (Tag-supported) and others where the spliced leader identity of the first gene is unknown (Predicted).

a The operon CBROPX0001 contains 8 genes in total. The first gene *(Cbr-rpb-6)* lacks a tag but has been confirmed by RT-PCR as SL1 spliced.

**Fig. 4.**
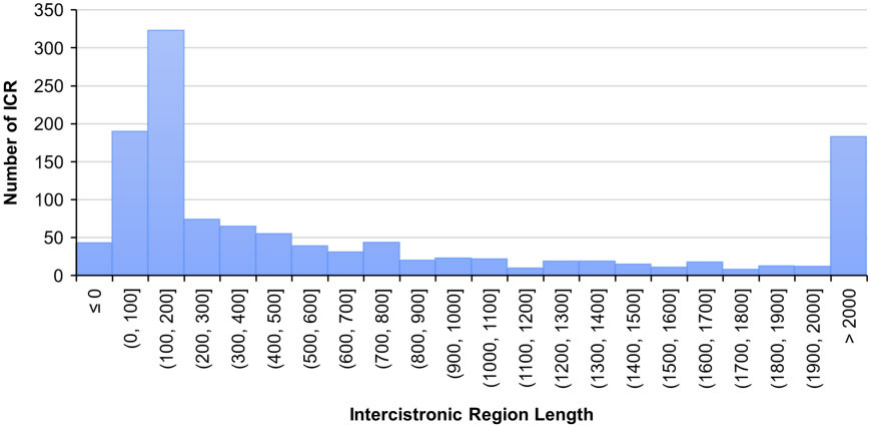
Frequencies of ICR lengths between SL2 and hybrid-spliced genes in operons. ICRs are sorted in bins of 100 nucleotides. For pairs of genes where the second gene is within the first gene, ICR is calculated as a negative value. For bin sizes, round brackets indicate exclusive bound, square brackets indicate inclusive bounds. Genes with larger than 2 kb ICRs are shown as a single peak.

**Fig. 5.**
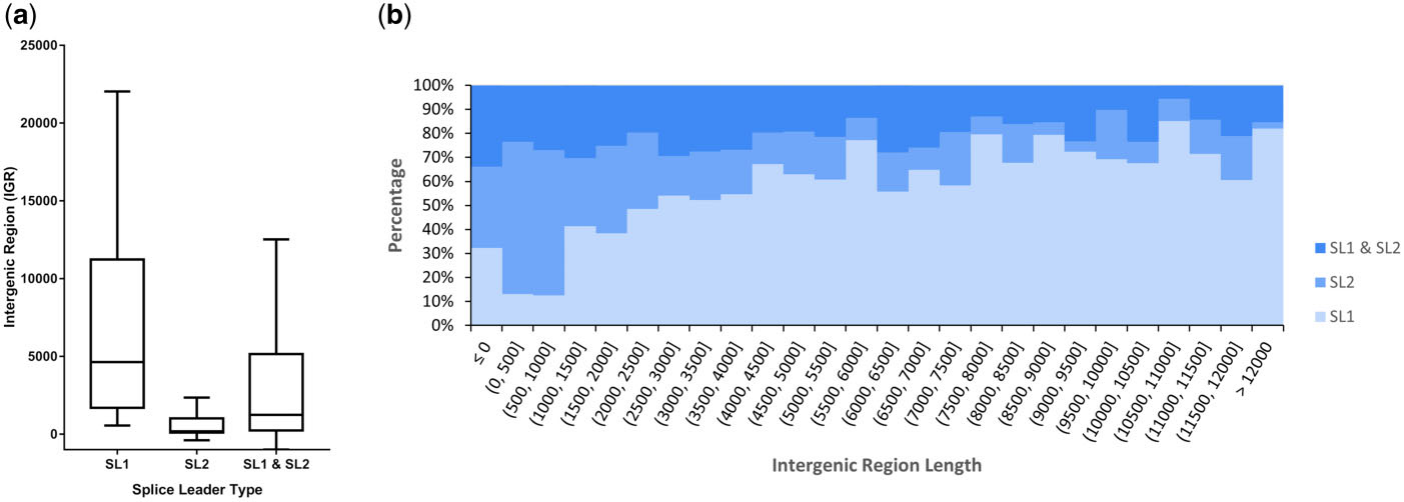
IGRs of genes identified by tag matches. a) Box plots show IGRs for SL1-spliced, SL2-spliced, and SL1/SL2-spliced genes. The inside line marks the median; lower, and upper lines represent the borders of the 25th and 75th quartile of the data sample, respectively. Whiskers enclose the 10–90% range of the data. b) 100% stacked columns of IGR length. Lengths are sorted in bins of 500 nucleotides. For pairs of genes where the second gene is overlapping or inside the first gene, length was calculated as a negative value. For bin sizes, round brackets indicate exclusive bound, square brackets indicate inclusive bounds.

In *C. elegans*, operon genes tend to be very closely spaced, typically having an ICR of <1 kb (Allen *et al.* 2011; Blumenthal *et al.* 2014). To examine whether the same is true in *C. briggsae*, we calculated ICRs and found that a vast majority of the genes (78%) are separated by <200 bp (Fig. 4). We also determined IGRs for SL1 and SL1/SL2 hybrid spliced genes discovered in our study. The results suggested that the IGR to the nearest gene upstream of SL2-spliced genes is smaller compared with those spliced with SL1 and SL1/SL2. While the SL2-spliced genes have a median distance of 180 bp, the medians of SL1 and SL1/SL2 spliced genes are 4,631 and 1,242 bp, respectively (Fig. 5a). Furthermore, as we would expect, genes with larger IGRs are more likely spliced with SL1 than SL2 or SL1/SL2 (Fig. 5b;Supplementary File 8) and are thus less likely to be part of the same operon.

#### Tag-supported operons

We examined the conservation of tag-supported operons in *C. elegans*. The analysis of orthologs helped define 3 distinct categories (Supplementary File 7). The 2 largest categories are named “exact match” and “partial match” operons (40% and 38%, respectively). Exact match operons consist entirely of *C. elegans* orthologs, whereas in partial match operons only some of the genes are conserved. The remaining one-fifth of operons define a third category, named “novel” (73). While a majority of these (61, 18%) consist of conserved genes whose orthologs are not present in *C. elegans* operons, others (12, 4%) consist of divergent, *C. briggsae*-specific genes.

**Fig. 6.**
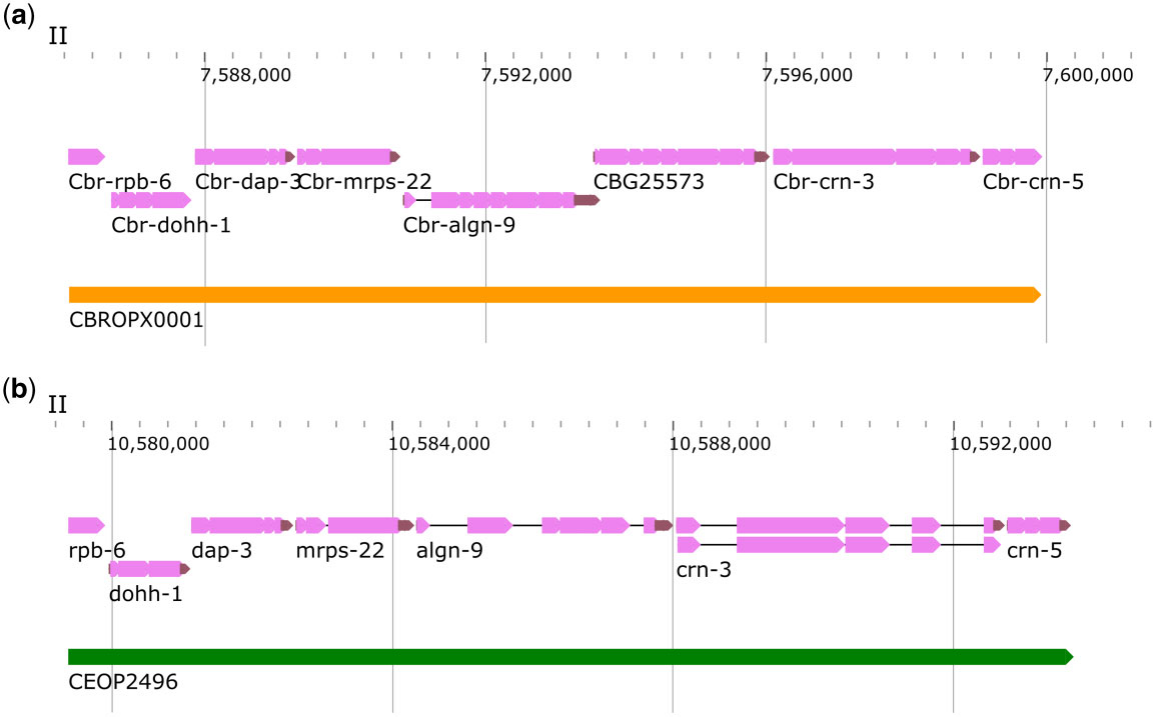
Genomic regions of *C. briggsae* CBROPX0001 and *C. elegans* CEOP2496. a) CBROPX0001 contains 8 genes. b) Homologous *C. elegans* operon CEOP2496 contains 7 genes. Genomic feature visualizations in this and subsequent figures are modified versions of images obtained from WormBase Jbrowse 2 (https://wormbase.org/tools/genome/jbrowse2/index.html).

The largest *C. briggsae* operons (CBROPX0001) was found to consist of 7 genes, 6 of which (*CBG25571, CBG03062, CBG25572, CBG03061, CBG03060, CBG03059*) are conserved in *C. elegans* and are part of the orthologous operon CEOP2496. The fifth gene in CBROPX0001 (*CBG25573*) does not appear to have a *C. elegans* ortholog. Syntenic alignments revealed that *CBG25573* is conserved in *C. brenneri*, suggesting that the gene may have been lost in the *C. elegans* lineage (Supplementary Fig. 4). While we did not recover a tag for *Cbr-rpb-6 (CBG03063)*, whose ortholog is the first gene in CEOP2496, we hypothesized that it is part of *C. briggsae* operon CBROPX0001 based on the distance from its neighbor *CBG25571* (195 bp) (Fig. 6). RT-PCR experiments revealed that *Cbr-rpb-6* is spliced to the SL1 leader sequence (Supplementary Fig. 5), thereby providing support to our hypothesis. We conclude that CBROPX0001 contains 8 genes.

**Fig. 7.**
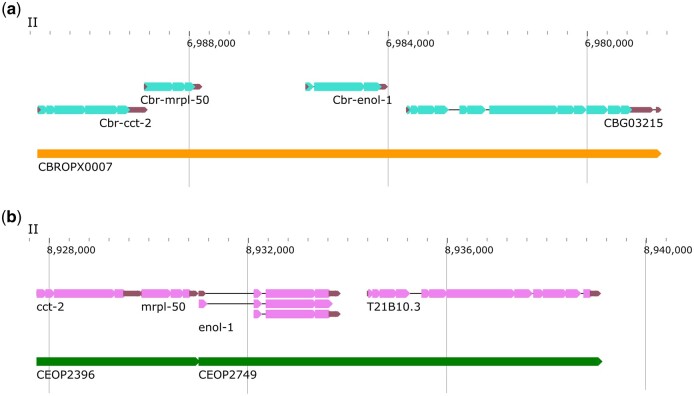
*C. briggsae* operon CBROPX0007. a) A cluster of 4 genes that define CBROPX0007. b) The orthologs of the 4 genes are split between 2 *C. elegans* operons CEOP2396 and CEOP2749.

A few operons were manually updated. For example, CBROPX0002 was split based on consensus SL, ICR between the genes (4,759), and homology information in *C. elegans*, resulting in 2 different operons: CBROP0132 (*CBG01778, CBG31146, CBG01779*) and CBROP0133 (*CBG01783, CBG01784*). In a different case, CBROPX0007 is predicted to consist of 4 genes (*CBG03212*, *CBG03213*, *CBG03214*, and *CBG03215*) (Supplementary Fig. 6). The *C. elegans* orthologs of these genes constitute 2 distinct operons (CEOP2396 and CEOP2749) (Fig. 7). While the ICR between *CBG03213* and *CBG03214* is larger than 2 kb, all downstream genes in CBROPX0007 are either SL2 or SL1/SL2 spliced. Further experiments are needed to validate the structure of CBROPX0007. Table 7 lists the updated numbers of operons in each category.

**Table 7.** Tag-supported operons in *C. briggsae.*

Operon type	Number (% of total)
Fully conserved operons (Exact match)	132 (40.1%)
Partially conserved operons (Partial match)	128 (38%)
Novel operons	73 (21.9%)
consisting of both divergent genes as well as orthologs that are not part of *C. elegans* operons	61 (18.3%)
consisting entirely of divergent genes	12 (3.6%)
**Total**	**333**

Exact match operons are conserved between *C. briggsae* and *C. elegans*. Partially conserved operons may contain some but not all orthologs that are part of corresponding *C. elegans* operons. Novel operons may contain *C. elegans* orthologs and divergent, *C. briggsae*-specific, genes.

**Fig. 8.**
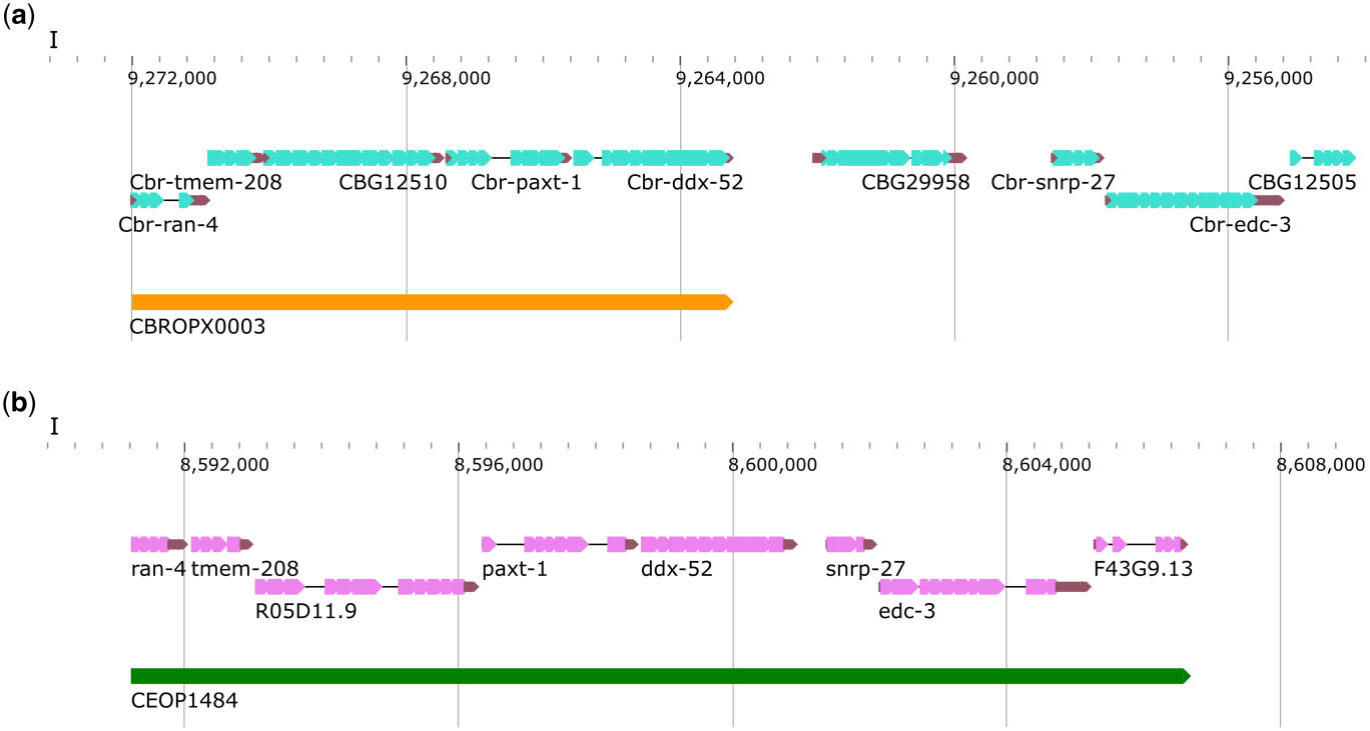
Partially conserved operon and its *C. elegans* ortholog. a) CBROPX0003 is an example of a partially conserved operon identified in this study. b) CEOP1484, *C. elegans* operon orthologous to *C. briggsae* operon CBROPX0003.

We also analyzed partially conserved operons in some detail. While all of these contain *C. elegans* orthologs, their structures are not conserved. Specifically, the number of genes or some of the orthologs in corresponding operons differ between the 2 species (Supplementary File 7). Of the 128 such operons, 83 contain 2 or more conserved genes including 58 (70% of 83) with <1 kb ICR between every gene. One such operon (CBROPX0003) consists of 5 genes (Fig. 8). While the *C. elegans* operon CEOP1484 contains orthologs of all of these, CEOP1484 encompasses 3 additional genes.

Our tag searches identified 73 novel operons (Supplementary File 7). A majority of these (61, 84%) consist of a mix of conserved genes and those that lack orthologs in *C. elegans*. It is important to point out that none of the conserved genes are part of *C. elegans* operons. The other 12 (17%) operons consist entirely of genes that lack orthology in *C. elegans*. In 7 of these cases, ICRs are <1 kb, providing further support to the operon structures (Table 8).

**Table 8.** Novel *C. briggsae* operons identified in this study with ICRs of <1 kb.

*Caenorhabditis briggsae* operon	No. of genes	Gene names (ICR)
CBROPX0130	3	*CBG30062* (172) *CBG25686* (105) *CBG25687*
CBROPX0131	3	*CBG27303* (533) *CBG27302* (116) *CBG27301*
CBROPX0140	2	*CBG11551* (162) *CBG31489*
CBROPX0129	3	*CBG21606* (235) *CBG30457* (493) *CBG21605*
CBROPX0139	2	*CBG30329* (76) *CBG30328*

None of the genes in these operons have orthologs in *C. elegans*. The numbers in brackets refer to ICR.

To investigate whether the 12 novel operons are unique to *C. briggsae* or might be conserved, the analysis was extended to *C. nigoni*, a sister species to *C. briggsae* (Woodruff *et al.* 2010). For this, we manually searched 5′ upstream regions of orthologs with ≥90% sequence similarity to 5′ tags and splice acceptor sites of *C. briggsae* genes. The sequence searches revealed that 4 of the operons have orthologs in the same genomic order with highly similar splice site sequences and small ICRs (≥1,200 bp), suggesting that they are conserved (Supplementary File 11).

#### Predicted (nontag-supported) operons

**Fig. 9.**
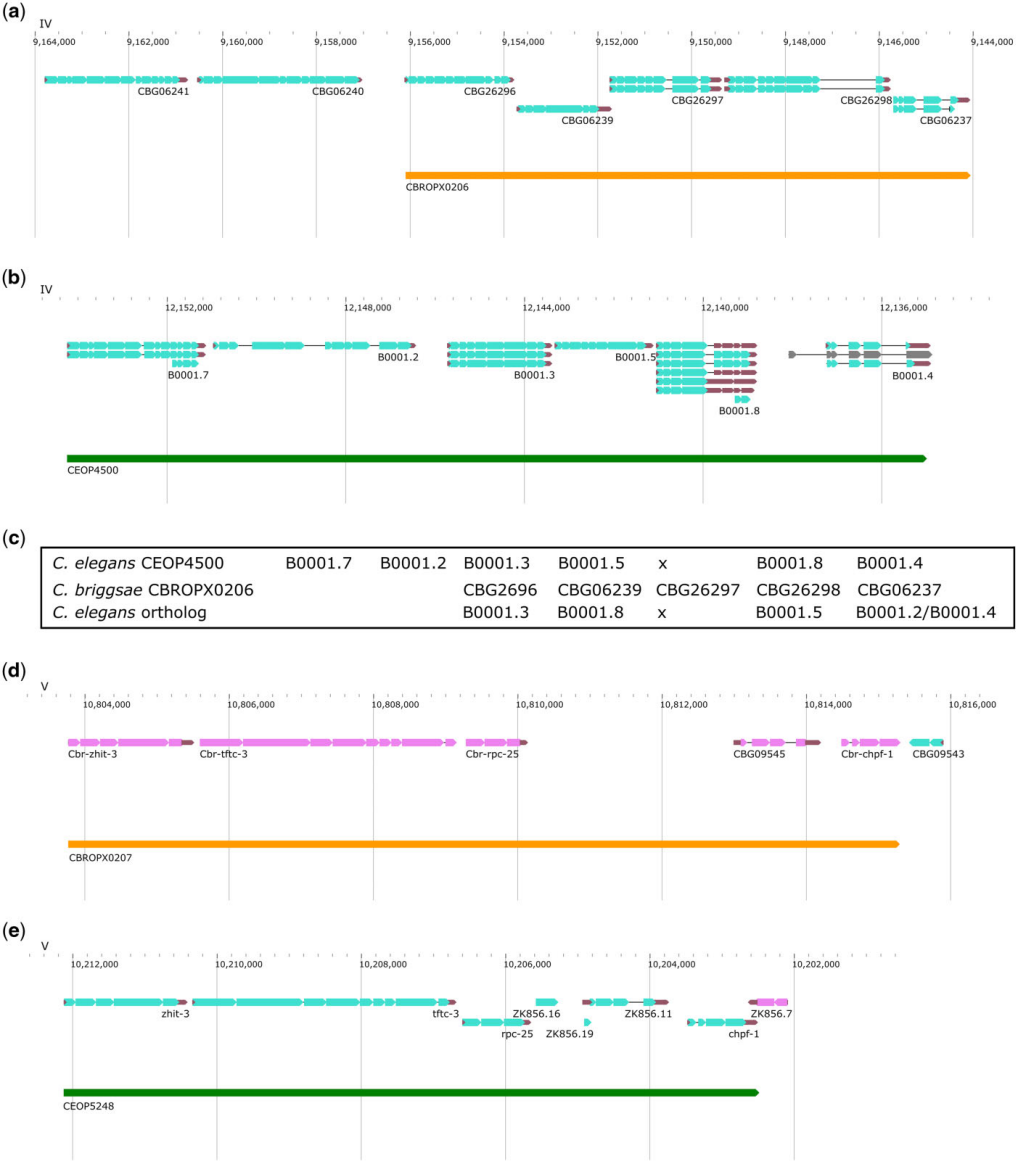
Two predicted operons in *C. briggsae* along with their *C. elegans* counterparts. a, b) CBROPX0206 with 5 genes and its orthologous operon CEOP4500 in *C. elegans*. Three genes are conserved between these 2 operons. c) The arrangement of genes in *C. elegans* operon CEOP4500 (row 1) and *C. briggsae* operon CBROPX0206 (row 2). The *C. elegans* orthologs of CBROPX0206 genes are shown in row 3. “x” denotes a missing ortholog. *CBG06237* is orthologous to both *B0001.2* and *B0001.4*. d, e) CBROPX0207 with 5 genes and its orthologous operon CEOP5428 with 7 genes. All 5 genes of the *C. briggsae* operon are conserved in CEOP5428. Two additional genes are present in CEOP5428.

We report 865 predicted operons (Supplementary File 7). While the downstream genes in these cases are spliced either with SL2 or SL1/SL2, the splicing status of the upstream gene is unknown. Most, if not all, of these are predicted to be genuine operons, especially those that are larger, i.e. consist of more than 2 genes. A comparison with *C. elegans* of 159 operons containing 3 or more genes revealed that 26 (16%) are fully conserved. A couple of examples include CBROPX0206 (5 genes) (Fig. 9, a and c) and CBROPX0207 (5 genes) (Fig. 9d). The corresponding *C. elegans* operons are CEOP4500 (6 genes) (Fig. 9, b and c) and CEOP5248 (7 genes), respectively (Fig. 9e). Comparison of genes in CBROPX0206 and CEOP4500 revealed that these share 4 orthologs. We observed 2 additional differences between CBROPX0206 and CEOP4500: the order of genes has changed and CBROPX0206 includes *CBG26297* which appears to lack a *C. elegans* ortholog (Fig. 9c). Given that *CBG06240* and *CBG36241* are immediately upstream of CBROPX0206 and their orthologs are part of CEOP4500, the *C. briggsae* operon may be extended to include both these genes. However, we have excluded these from our operon model in the absence of corresponding TEC-RED tags. The second example, CBROPX0207, contains 5 genes, all of which have orthologs in CEOP5248. However, the *C. elegans* operon contains 2 additional genes (*ZK856.16* and *ZK856.19*) which are not conserved in *C. briggsae.*

Uyar *et al.* (2012) had previously reported operons in *C. briggsae* based on RNA-seq experiments. A comparison with our dataset revealed partial or complete overlap with 195 operons (16%), including 15 that are identical (Table 9; Supplementary File 7). A lack of significant match between the 2 datasets is unexpected. One explanation would be that gene models and genome assembly have undergone substantial changes since the previous study.

**Table 9.** Comparison of TEC-RED-identified operons with Uyar *et al.* operons.

Match type	Operons matching with Uyar *et al.* study	High- confidence operons	Tag- supported operons	Predicted operons
Identical	15	15	4	11
Same start	33	13	8	20
Same end	23	11	7	12
Overlap	124	53	33	71

Operons compared between this study and Uyar *et al.* are divided into 4 categories. TEC-RED operons that contained the same set of genes, i.e. same start and end points, were marked “Identical.” TEC-RED operons that overlapped, but either contained more genes downstream or upstream were marked “Same start” or “Same end,” respectively. TEC-RED operons that partially overlapped in other ways were grouped into “Overlap.”

#### SL1-type operons

We also found 2 operons that contain only SL1-spliced genes. These genes are positioned directly adjacent to one another, with no space between them. The SL1-type operons have been described previously in *C. elegans* and shown to lack ICR (Williams *et al.* 1999). One of the *C. briggsae* SL1-type operons consists of 2 genes: CBROP0134 (*CBG16825*, *Cbr-vha-11*/*CBG16826*). Its *C. elegans* ortholog, CEOP4638, also consists of 2 genes. Another SL1-type operon identified by our study is CBROPX0001. Its *C. elegans* ortholog is CEOP2496. Interestingly, CBROPX0001 and CEOP2496 consist of more than 2 genes (Fig. 6). In the case of CEOP2496, the first 2 genes (*rpb-6* and *dohh-1*) are known to be spliced exclusively with SL1 (defined as SL1 operon) whereas the remaining downstream genes with SL2 or both SL1 and SL2.

**Fig. 10.**
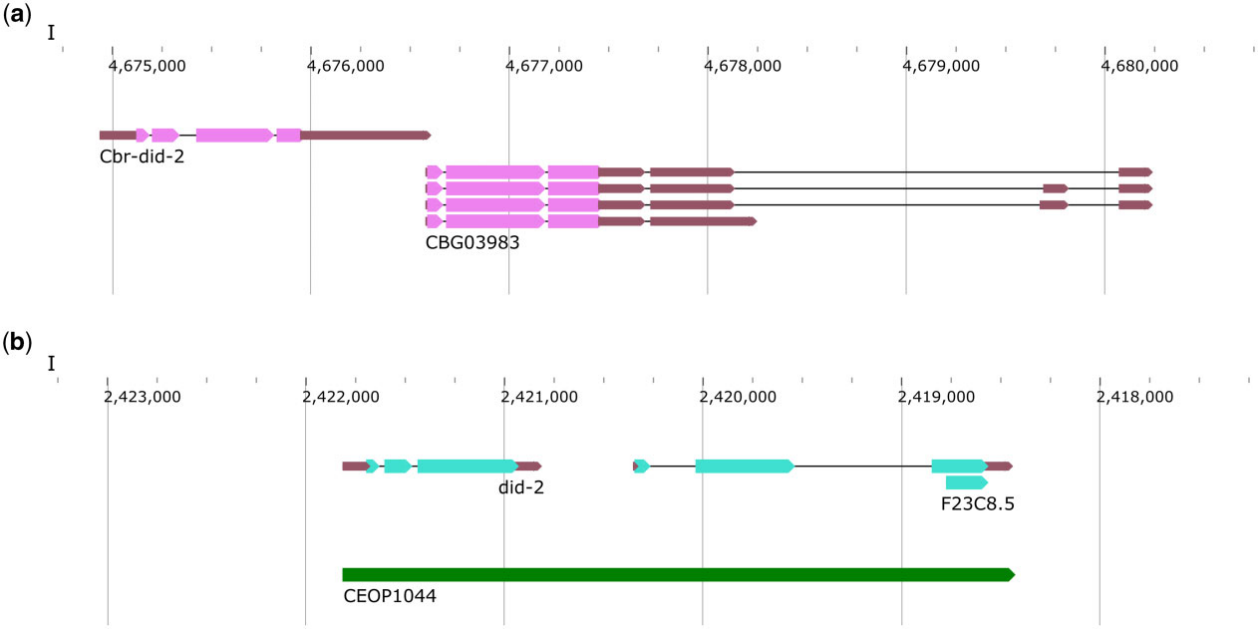
*C. briggsae* genes with a single base pair ICR. a) Both *CBG03984* and *CBG03983* are spliced with SL1 leader sequences and located 1 bp apart. b) *C. elegans* orthologs *did-2* and *F23C8.5*, respectively, are part of the operon CEOP1044.

There is also a potential SL1-type operon consisting of *CBG03984* and *CBG03983*. These 2 genes have a single base pair IGR (Fig. 10). Interestingly, the *C. elegans* orthologs, *F23C8.6* and *F23C8.5* (SL1 and SL1/SL2 spliced, respectively) are part of 1 operon, CEOP1044, with an ICR of more than 400 bp (Allen *et al.* 2011). More work is needed to determine whether the *C. briggsae* genes are indeed part of an SL1-type operon.

#### Caenorhabditis briggsae operons show enrichment of germline genes and highly expressed growth genes

Studies in *C. elegans* and *Pristionchuspacificus* have reported that germline genes are overrepresented in operons (Reinke and Cutter 2009; Sinha *et al.* 2014). We did a gene-association study in *C. briggsae* to examine a similar possibility. The results revealed a significant enrichment of germline genes in high confidence operons (*P* < 7.40E−98) (Supplementary File 9).

In addition to investigating germline genes, we performed GO term analysis of operon genes and found enrichment of terms associated with metabolic and biosynthesis processes. The pattern of enrichment was similar to what was observed with a *C. elegans* operon dataset (Supplementary File 9). We also found enrichment of growth-related genes, as in *C. elegans*, specifically, female gamete generation (GO:0007292), embryo development ending in birth or egg hatching (GO: 0009792), reproduction (GO:0000003), and embryo development (GO:0009790) (Zaslaver *et al.* 2011). It is important to point out that while GO terms are similar in both species, *C. briggsae* operon genes associated with specific processes are not always the orthologs of *C. elegans* gene sets. We therefore conclude that functions of operon genes are conserved even if specific genes are not.

## Discussion

**Fig. 11.**
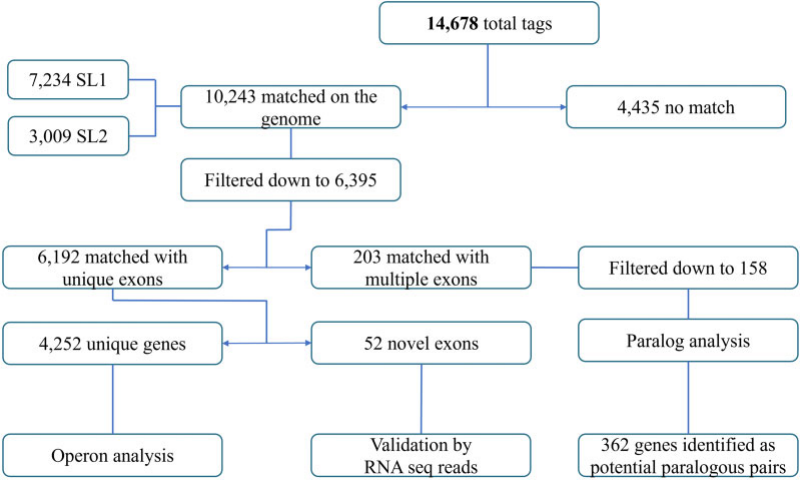
An overview of TEC-RED analysis in *C. briggsae*. The 5′ sequence tags were used to identify exons and genes. Further analysis resulted in the discovery of operons, paralogs, and novel exons.

This paper reports major improvements in the genome annotation of *C. briggsae*. We recovered 10,243 unique 5′ end tags with matches in the genome, of which 6,395 correspond to SL1 and SL2 spliced exons and provide support to the existence of 4,252 unique *trans*-spliced genes. Another 362 genes have been identified as paralogs, including 42 for which the paralogous relationship is supported by WormBase annotation. We also report 52 novel exons that may define new genes or exons of existing genes. Figure 11 provides a global overview of sequences identified in our analysis.

In *C. elegans*, 84% of all genes are spliced to leader sequences (Tourasse *et al.* 2017). If the percentage is comparable in *C. briggsae*, then our work has resulted in the identification of roughly one-quarter of all *trans*-spliced genes in this species. Further analysis has revealed that two-thirds of all *C. briggsae* genes are spliced with SL1, while the rest are split evenly between SL2 and SL1/SL2 hybrid sequences (65% SL1, 18% SL2, and 18% SL1/SL2). Assuming that the TEC-RED method is unbiased in regard to the recovery of SL1 and SL2 spliced transcript tags, the proportion of spliced genes in *C. briggsae* differs from those in *C. elegans* as reported by Allen *et al.* (2011) (82% SL1, 12% SL2, and 8% SL1/SL2). Additionally, 14 genes were found to be spliced to leader sequences only in *C. briggsae* and not in *C. elegans*. More work is needed to determine if *trans*-splicing of these genes has indeed diverged between the 2 species.

Our analysis revealed that most of the genes identified by unique tag matches are represented by a single transcript (94.8%) and very few (5.2%) by multiple transcripts. Studies in *C. elegans* have reported roughly 18% of genes giving rise to multiple isoforms (Wang *et al.* 2010; Spieth *et al.* 2014), although this number is predicted to be as high as 25% (Ramani *et al.* 2011; Zahler 2012). Considering this, along with the fact that our experiments captured only a partial set of all spliced genes, the actual proportion of genes with multiple transcripts in *C. briggsae* is likely to be much higher. Among other things, it was found that 77.8% of the genes in our study have 5′ start sites that match with those annotated by WormBase. The remaining ones were considered mispredictions, most of which were major mispredictions (15.7%) as 5′ start sites in these cases map anywhere between 20 bp and 3 kb away from known locations. We also found 52 new, previously unreported exons that map more than 3 kb upstream to the nearest exon of existing genes, and potentially include some that define 5′ start sites of new genes.

Several approaches were taken to validate tag-based gene models. One involved comparing results with those obtained using an older genome annotation (WS176 gff) file. The findings revealed that a total of 858 genes for which 5′ ends were correctly annotated in WS276 were mispredicted or absent in the older version, which demonstrates that our data can help improve start sites of many *C. briggsae* genes. Another approach involved comparing 5′ ends of some of the genes with those of *C. elegans* orthologs. Of the 21 alternate start sites and 50 major mispredicted start sites analyzed, 38% and 34%, respectively, are supported by *C. elegans* transcripts. Finally, we examined the 52 newly discovered exons and found that 37% of these are supported by RNA-seq data in WormBase. The above 3 validations provide significant support to the accuracy of our analysis of expressed transcripts in *C. briggsae*.

The identification of genes spliced with leader sequences in *C. briggsae* allowed us to curate operons and study their conservation. Even though the operon-based organization of genes in *C. elegans* and *C. briggsae* is similar to those found in bacteria and archaea, work in *C. elegans* has shown that worm operons have no ancestral relationship with prokaryotes and appear to have evolved independently within the nematode phylum (Blumenthal 2004; Qian and Zhang 2008). We identified a total of 1,198 operons, of which 28% consist entirely of tag-supported genes. Of the remaining operons with partial tag support, 159 contain 3 or more genes. Combined with the fully tag-supported operons, this totals 492 operons in *C. briggsae* with a high degree of confidence.

Previously, operons in *C. elegans* were annotated by using RNA seq datasets (Allen *et al.* 2011; Tourasse *et al.* 2017). These studies used established criteria of SL type, location of ATG, and intergenic distances. We have used a similar approach with one major difference, i.e. our method of finding *trans-*spliced genes is based on short 5′ tags and not RNA-seq reads. To further confirm the operons identified by TEC-RED, we compared them with those annotated in Uyar *et al.* (2012) study and found 15 to be identical, with a further 180 overlapping partially. Together, this accounts for 16% of all operons (tag-supported and predicted) discovered in this study. The remaining TEC-RED operons are being reported for the first time. In the future, approaches such as RNA-seq may be used to further extend our findings.

Comparison of tag-supported operons with *C. elegans* revealed that 132 (40%) are conserved, with the remainder being partially conserved (128, 39%) and novel (73, 21%). A subset of novel operons (12, 17%) consists entirely of genes that lack *C. elegans* orthologs. Further comparisons with *C. nigoni* revealed that 4 of the 12 are likely to be conserved, suggesting that these might have arisen in the common ancestor shared between *C. briggsae* and *C. nigoni*. Whether the remaining 8 are unique to *C. briggsae* requires more analysis. Along with the above-mentioned operons, we also uncovered 2 conserved SL1-type operons. Together, these data demonstrate that while many of the operons are conserved, there are substantial differences between the 2 species. The findings represent the first comprehensive analysis of operons in *C. briggsae*.

In conclusion, the data presented in this study have significantly improved the annotation of the *C. briggsae* genome by validating existing gene models, refining start sites of many genes, identifying novel gene exons, alternate transcripts, and by providing a comprehensive analysis of operons and paralogous gene sets. While the majority of the genes and operons are conserved in *C. elegans*, our work has also revealed substantial differences between the 2 species. The improvements to the genome annotation reported here are expected to strengthen *C. briggsae* as a model for comparative and evolutionary studies.

## Data availability

The data underlying this article are available in the article and in its online supplementary material.

Supplemental material is available at *G3* online.

## Supplementary Material

jkac101_Supplementary_Data_File_1

jkac101_Supplementary_Data_File_2

jkac101_Supplementary_Data_File_3

jkac101_Supplementary_Data_File_4

jkac101_Supplementary_Data_File_5

jkac101_Supplementary_Data_File_6

jkac101_Supplementary_Data_File_7

jkac101_Supplementary_Data_File_8

jkac101_Supplementary_Data_File_9

jkac101_Supplementary_Data_File_10

jkac101_Supplementary_Data_File_11

jkac101_Supplementary_Material
