# Supplementary material for: Genome annotation of Caenorhabditis briggsae by TEC-RED identifies new exons, paralogs, and conserved and novel operons
Source: G3 (Bethesda). 2022 Apr 29;12(7):jkac101. doi: 10.1093/g3journal/jkac101 (PMC9258526; doi:10.1093/g3journal/jkac101)
Supplement: jkac101_Supplementary_Material [file jkac101_supplementary_material.docx]

**SUPPLEMENTARY MATERIALS**

**Jhaveri and van den Berg et al. Genome annotation of *Caenorhabditis briggsae* by TEC-RED identifies new exons, paralogs, and conserved and novel operons**

**SUPPLEMENTARY DATA FILES (Microsoft Excel spreadsheets)**

| **File name** | **Description** |
| --- | --- |
| Supplementary data file 1 | Exons identified in our analysis |
| Supplementary data file 2 | Unique genes identified |
| Supplementary data file 3 | Genes uniquely spliced in *C. briggsae* |
| Supplementary data file 4 | Validation based on overlap with WS176 |
| Supplementary data file 5 | New exons identified by our study |
| Supplementary data file 6 | Manual curation of 1b and major mispredictions based on *C. elegans* orthologs |
| Supplementary data file 7 | List of operons |
| Supplementary data file 8 | Intergenic region values |
| Supplementary data file 9 | Germline genes present in operons and GO analysis |
| Supplementary data file 10 | Proposed paralog sets |
| Supplementary data file 11 | Matches to *C. briggsae* novel operons in *C. nigoni* |

**SUPPLEMENTARY TABLES**

**Supplementary Table 1:** Primers used to generate Biotin-RT-PCR products

| **Primers** | **Sequence (5’ to 3’)** |
| --- | --- |
| RT primer | GTGATGTCTCGAGTAGTTCGAAATGGCC (T)22 |
| 5’ SL1-*Bpm*I RT-PCR primer | Biotin/ AGACGCAAGGTTTAATTACCCAAGCTGGAG |
| 5’ SL2-*Bpm*I RT-PCR primer | Biotin/ AGACGCAAGGTTTTAACCCAGTTACTGGAG |
| 3’ RT-PCR primer | GAGGTGATGTCTCGAGTAGTTCGAAATGGC |

**Supplementary Table 2:** PCR primers used to generate mono-tags from the 5’ biotin-adaptor DNA fragments

| **Primers** | **Sequence (5’ to 3’)** |
| --- | --- |
| 5’ SL1-*Xho*I primer | AGACGCAAGGTTTAATTACCCAAGCTCGAG |
| 5’ SL2-*Xho*I primer | AGACGCAAGGTTTTAACCCAGTTACTCGAG |
| 3’ for adaptor 1 (*Kpn*I) | CTATAGGGCTCAAAGATGACGAGAGGA |
| 3’ for adaptor 2 (*Hind*III) | CAAGATTCTCACGACGATGTTCGGAGT |
| 3’ for adaptor 3 (*Eag*I) | TGAAGATTGCACAGAGGAGAGACCGCT |
| 3’ for adaptor 4 (*Sac*I) | CAGTTGGAATGAATGAAGCTATACCAT |
| 3’ for adaptor 5 (*Mlu*I) | CTAGTATACGTTCTAGTATCAGAGGAA |
| 3’ for adaptor 6 (*Nhe*I) | TCTTGCAGTGATTAGCGTCAGTGCCTG |

**Supplementary Table 3:** Adaptors used for ligation onto *Bpm*I-digested, 5’ biotin-DNA fragments

| **Adapter** | **Sequence (5’ to 3’)** | **Sequence (3’ to 5’)** |
| --- | --- | --- |
| Adapter 1 (*Kpn*I) | CTATAGGGCTCAAAGATGACGAGAGGAGGTACC | TGCTCTCCTCCATGG |
| Adapter 2 (*Hind*III) | CAAGATTCTCACGACGATGTTCGGAGTAAGCTT | CAAGCCTCATTCGAA |
| Adapter 3 (*Eag*I) | TGAAGATTGCACAGAGGAGAGACCGCTCGGCCG | CTCTGGCGAGCCGGC |
| Adapter 4 (*Sac*I) | CAGTTGGAATGAATGAAGCTATACCATGAGCTC | GATATGGTACTCGAG |
| Adapter 5 (*Mlu*I) | CTAGTATACGTTCTAGTATCAGAGGAAACGCGT | AGTCTCCTTTGCGCA |
| Adapter 6 (*Nhe*I) | TCTTGCAGTGATTAGCGTCAGTGCCTGGCTAGC | GTCACGGACCGATCG |

**Supplementary Table 4:** Chromosomal locations of 4,252 unique genes identified by TEC-RED. Chr: Chromosome, Un: unmapped genomic region.

| **Chr** | **Total gene count** | **SL1 genes** | **Fraction** | **Density** | **SL2 genes** | **Fraction** | **Density** | **SL1/SL2 genes** | **Fraction** | **Density** | **Chr length (Mb)^*^** |
| --- | --- | --- | --- | --- | --- | --- | --- | --- | --- | --- | --- |
| I | 763 | 447 | 16.26 | 28.93 | 158 | 21.27 | 10.23 | 158 | 20.79 | 10.23 | 15.45 |
| II | 752 | 473 | 17.21 | 28.46 | 147 | 19.78 | 8.84 | 132 | 17.37 | 7.94 | 16.62 |
| III | 751 | 444 | 16.15 | 30.47 | 145 | 19.52 | 9.95 | 162 | 21.32 | 11.12 | 14.57 |
| IV | 717 | 447 | 16.26 | 25.57 | 133 | 17.90 | 7.61 | 137 | 18.03 | 7.84 | 17.48 |
| V | 749 | 534 | 19.43 | 27.40 | 105 | 14.13 | 5.39 | 110 | 14.47 | 5.64 | 19.49 |
| X | 515 | 400 | 14.55 | 18.57 | 54 | 7.27 | 2.51 | 61 | 8.03 | 2.83 | 21.54 |
| Un | 5 | 4 |  |  | 1 |  |  | 0 |  |  |  |
|  | 4252 | 2749 |  | 26.14 | 743 |  | 7.07 | 760 |  | 7.23 | 105.15 |

^*^Ross, J. A., D. C. Koboldt, J. E. Staisch, H. M. Chamberlin, B. P. Gupta et al., 2011 *Caenorhabditis briggsae* recombinant inbred line genotypes reveal inter-strain incompatibility and the evolution of recombination. PLoS Genet 7: e1002174.

**Supplementary Table 5:** Intergenic distances of selected Category 3 genes that are less than 10 kb apart.

| **Adjacent genes identified by tags** | **IGR** | **BLAST alignment** | ***C. elegans* orthologs** | ***C. briggsae* gene orientation** |
| --- | --- | --- | --- | --- |
| *CBG25816, CBG00473* | 2,903 bp | Yes | None | Opposite |
| *CBG08766, CBG08768a* | 578 bp | No^*^ | *F25E5.8* and *nhr-117* | Opposite |
| *CBG25203, CBG29819* | 2,251 bp | No | *F59A3.2* and *ubl-5* | Opposite |
| *CBG26374, CBG05421* | 3,564 bp | No^*^ | None, *fan-1* | Same |
| *CBG26845, CBG26846* | 8,559 bp | Yes | None | Same |

**^*^**BLAST match showed some similarity in a very small 5’ region.

**SUPPLEMENTARY FIGURES**

**Supplementary Figure 1:** Flowchart of steps used to analyze 5’ tag sequences and genes.


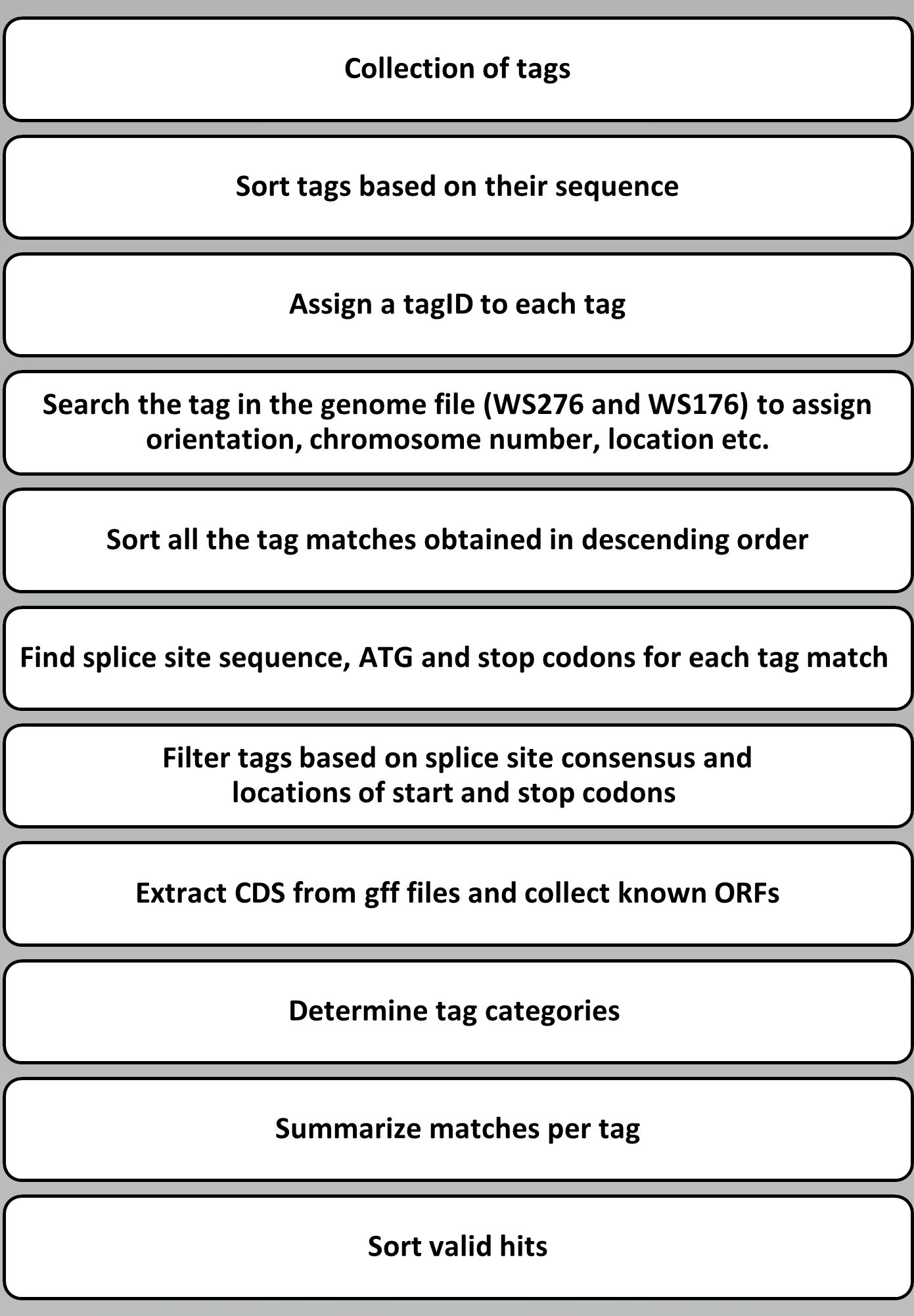


**Supplementary Figure 2:** Selected examples of novel exons identified by tags that are supported by WormBase RNASeq data. The top track of the genome browser shows currently curated genes. Second track shows alignments of short read sequences from all available RNASeq projects on WormBase. The number of reads has been normalized by averaging over the number of libraries. The height of reads boxes indicates the relative score of the feature. The bottom track shows a TEC-RED tag binding at a genome location predicted to contain the 5’ start site of a new exon. **(A)** New exon between *CBG12426b.1* and *CBG12425b.1*. **(B)** A new exon inside *CBG03270a.1*.


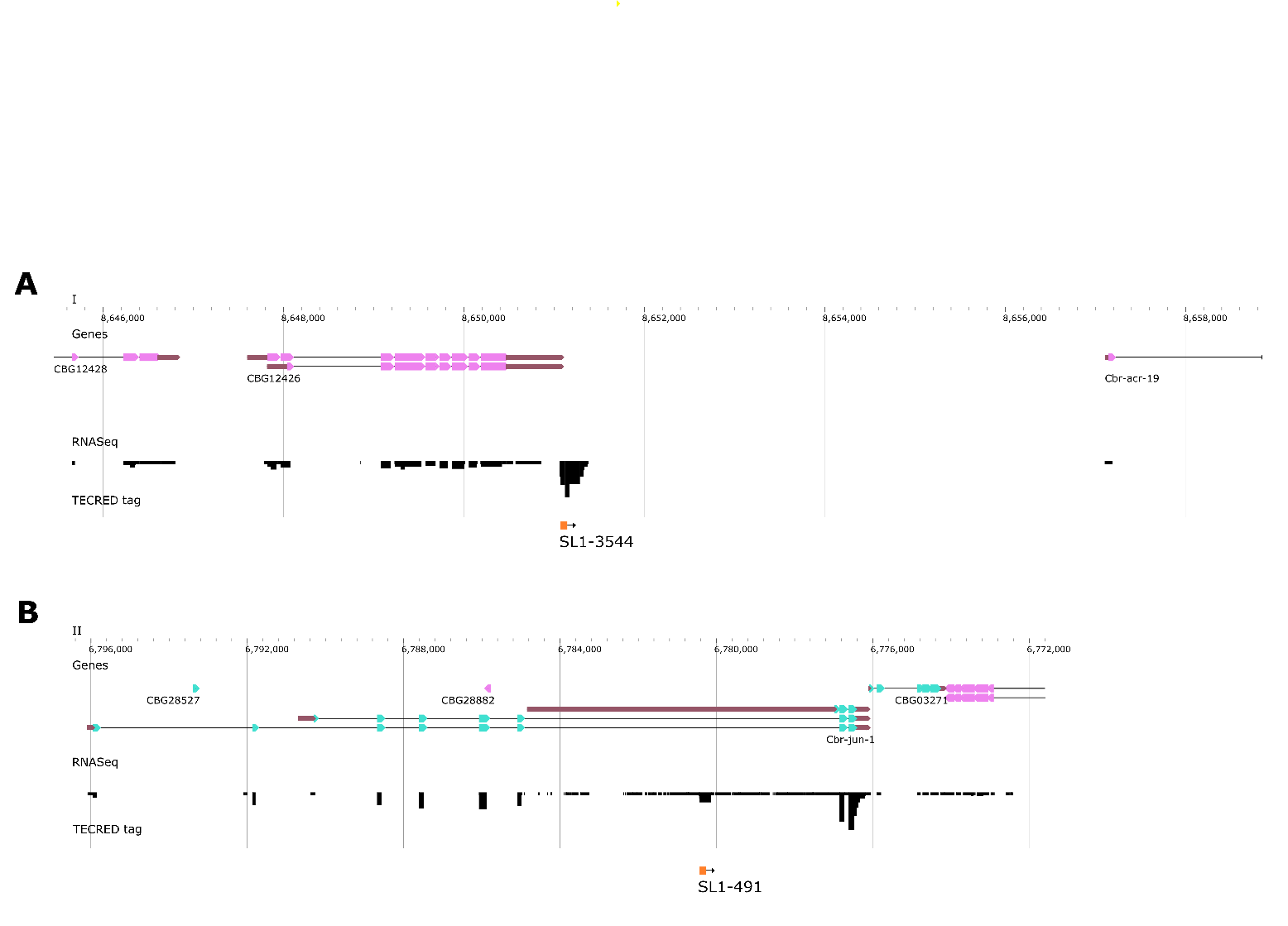


**Supplementary Figure 3:** An example of the 1b category in *Cbr-cdf-1* in WormBase Jbrowse genome browser. The top track shows curated gene *Cbr-cdf-1*. The middle track shows the *C. elegans* *C15B12.7a.1* (*cdf-1*) gene model, which is indicated in orange. The bottom track shows a category 1b TEC-RED tag binding at the 5’ start site of exon 2 of *Cbr-cdf-1*. The *C. elegans* gene model supports the 5’ start site of a new transcript variant for *Cbr-cdf-1*.


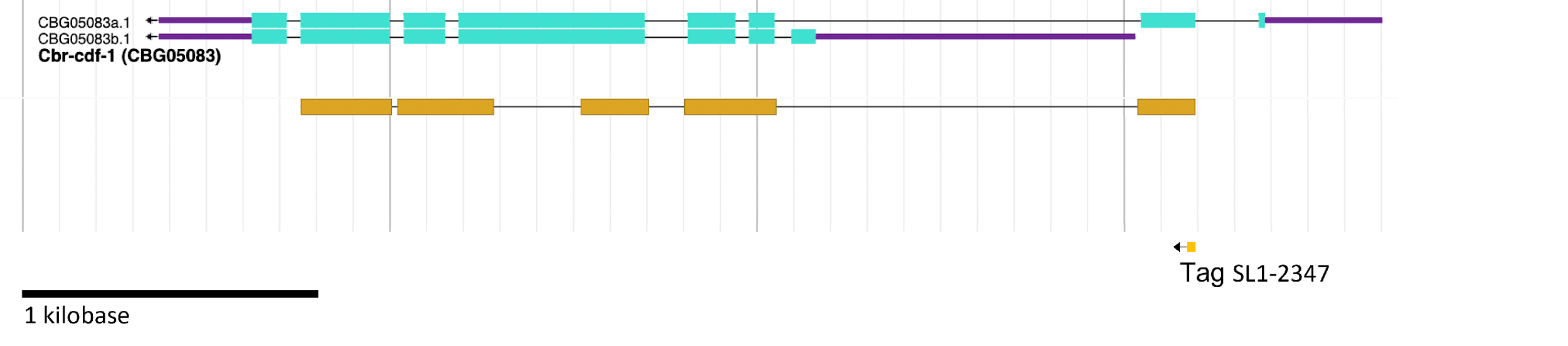


**Supplementary Figure 4.** A screenshot of the WormBase synteny browser showing the genomic region of *C. briggsae* operon CBROPX0001, and the corresponding regions in *C. brenneri*, *C. elegans* and *C. briggsae*.

**
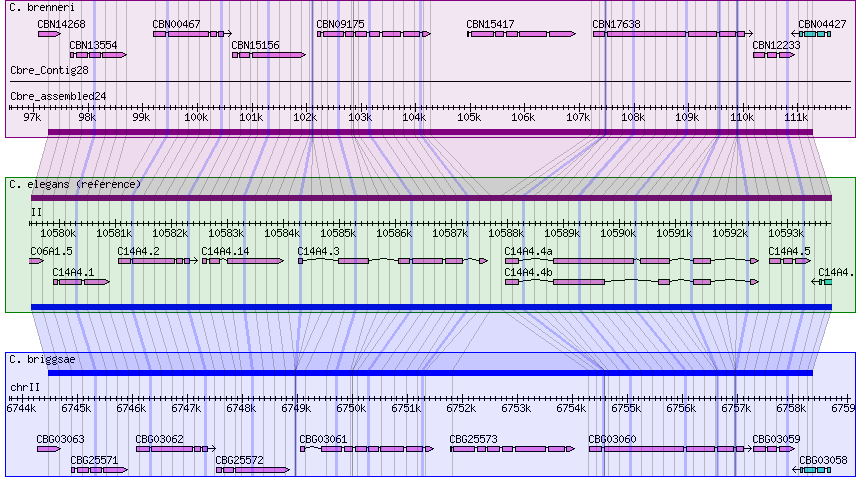
**

**Supplementary figure 5:** The open reading frame of *Cbr-rpb-6* containing a single exon. The gene is spliced to SL1 leader sequence as confirmed by RT-PCR. Additionally, it may be spliced to SL2 (dotted box) based on the low transcript abundance but needs to be further confirmed.

**
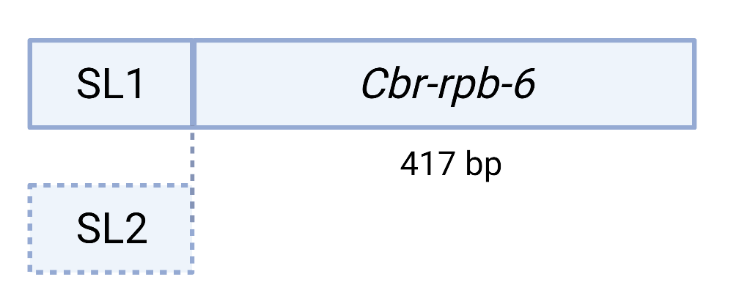
**

**Supplementary figure 6:** A screenshot of the WormBase synteny browser showing the genomic region consisting of a cluster of four *C. briggsae* genes that define the CBROPX0007 operon and corresponding region in *C. elegans*.

**
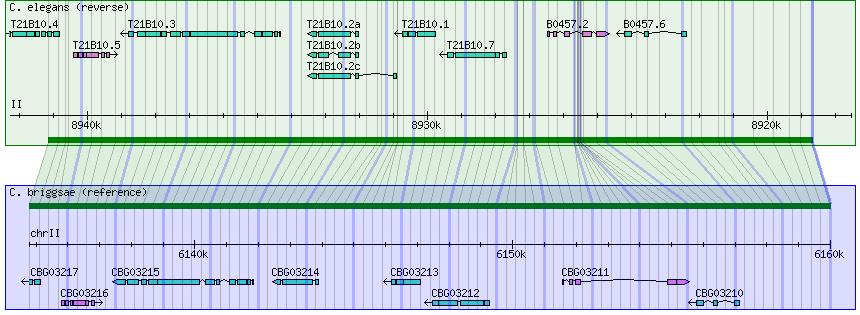
**
